# Lance-Adams Syndrome in the Intensive Care Unit: A Case Report

**DOI:** 10.7759/cureus.58241

**Published:** 2024-04-14

**Authors:** Diego Angeles-Sistac, Marta Izura-Gomez, Ainara Barguilla-Arribas, Alba Sierra-Marcos, Indalecio Moran-Chorro

**Affiliations:** 1 Critical Care Medicine, Hospital de la Santa Creu i Sant Pau, Barcelona, ESP; 2 Neurology, Hospital de la Santa Creu i Sant Pau, Barcelona, ESP

**Keywords:** seizure phenomenology, movement disorders and tremors, medical intensive care unit (micu), acute cerebral hypoxia, neurocritical care unit, cardiac arrest, case report, post-hypoxic myoclonus, acute post-hypoxic myoclonus, lance-adams syndrome (las)

## Abstract

Lance-Adams syndrome (LAS), or chronic post-hypoxic myoclonus, is a myoclonic disorder following acute cerebral hypoxia after successful cardiopulmonary resuscitation (CPR). LAS is distinct from acute post-hypoxic myoclonus (acute PHM), presenting with myoclonic jerks and cerebellar ataxia after regaining consciousness. However, the overlap at the onset complicates differentiation and may lead to the withdrawal of life-sustaining measures, especially in sedated ICU patients.

The presented case involves a 77-year-old male diagnosed with LAS post-CPR. Despite the presence of early myoclonic jerks EEG, laboratory testing, and neuroimaging showed no definitive proof of irreversible neurological damage. Once diagnosed, treatment involved sequential antiseizure medications and physical therapy when the patient achieved full consciousness. However, the patient ultimately faced severe disabilities and was unable to recover.

This case report emphasizes the importance of limiting sedation, comprehensive clinical examination, and the use of complementary tests when no definitive proof of irreversible neurological damage is present after acute cerebral hypoxia. While LAS has a better vital prognosis than acute PHM, it is associated with poor neurofunctional recovery and chronic disability in most cases. Further research is essential for evidence-based management.

## Introduction

The increased availability of out-of-hospital emergency services and technological advancements in intensive care units (ICU) have resulted in higher survival rates for patients experiencing cardiorespiratory arrest. However, individuals who undergo significant hypoxic events may develop long-lasting or fatal neurological complications. In this context, there are two main myoclonic syndromes associated with acute cerebral hypoxia: acute post-hypoxic myoclonus (acute PHM) and Lance-Adams syndrome (LAS) also referred to as chronic post-hypoxic myoclonus (chronic PHM) [1]. Due to the different clinical and prognostic implications associated with each one, it is important to differentiate between these two syndromes.

Acute PHM usually appears during the first 24 hours of hypoxic injury, and the development of multifocal myoclonus may take up to three days. This syndrome is characterized by generalized myoclonic jerks in patients who are deeply comatose [1-3]. Myoclonic seizures at any time after return to spontaneous circulation (ROSC) are considered to reliably indicate a poor prognosis. Acute PHM predicts a permanent vegetative state or death in more than 90% of survivors [2,4].

Chronic PHM was described by Lance Adams in 1963 after reporting four cases of intention or action myoclonus along with dysarthria and ataxia, which appeared a few days after cardiac arrest in patients who regained consciousness [5]. LAS is a rare complication of successful cardiopulmonary resuscitation, with a small number of case reports worldwide [6,7]. Most cases of LAS have been reported after respiratory arrest followed by anesthesia, surgical accidents, and asthmatic crisis, while fewer cases of LAS have been reported associated with cardiac arrest of a purely cardiological origin. Other uncommon etiologies of LAS include myocarditis and even hypoxia associated with coronavirus disease 2019 (COVID-19) infection without cardiac arrest [2,3,8].

It is crucial to highlight that the single most important distinguishing feature between acute and chronic PHM is the presence or absence of coma, LAS patients persist with myoclonic jerks and cerebellar ataxia after regaining consciousness [2,7]. LAS can be easily misdiagnosed since its clinical features may overlap with acute PHM, especially if myoclonus occurs in the first hours of hypoxia while the patient is still in a coma. LAS may develop within a few days or even months after the hypoxic event, and while acute PHM is present in a higher proportion of patients who develop LAS later, not all patients with LAS suffer from acute PHM before. Additionally, it is difficult to perform an accurate neurological examination and evaluate myoclonus in this type of patients because they are usually under the effects of sedatives or neuromuscular blocking agents [1,6,9]. While LAS has a better vital prognosis than acute PHM, it is associated with poor neurofunctional recovery and chronic disability in most cases [6]. LAS must be suspected in comatose patients experiencing myoclonic jerks if complementary neurological tests show no abnormalities.

We present a detailed case of an older patient who presented with early myoclonic jerks (less than 24 hours) after successful cardiopulmonary resuscitation (CPR) caused by malignant arrhythmia of unknown origin. All neurological testing were normal, he remained comatose for almost 20 days in the ICU but still exhibited myoclonic jerks after regaining consciousness and, therefore, he was diagnosed as having LAS.

## Case presentation

A 77-year-old male patient, with a medical history of hypertension, diabetes, hypothyroidism, and Child B score hepatic cirrhosis. Ongoing treatment included levothyroxine, olmesartan, amlodipine, thiazides, insulin, metformin, sertraline and simvastatin. He was admitted to our ICU with a Glasgow Coma Scale (GCS) of 3 in the absence of sedation. The patient experienced a witnessed cardiac arrest while on the street and received 10 minutes of basic cardiopulmonary resuscitation (CPR) until arrival of the emergency service, at which point a return of spontaneous circulation (ROSC) was noted and the patient was intubated. Upon arrival at the emergency department, the patient remained with the same GCS. An electrocardiogram (ECG) revealed the presence of a previously unidentified complete left bundle branch block. Initial echocardiography and computerized tomography (CT) of head and chest did not show any notable abnormalities.

During the initial 24-hour period of ICU admission, the patient exhibited myoclonic jerks involving face, shoulders and distal upper extremities; however, no corresponding epileptiform activity (EA) was recorded during various sequential electroencephalograms (EEG). This myoclonic activity persisted after starting intravenous (IV) levetiracetam at a dosage of 1 g every 12 hours. Consequently, sedation starting first with propofol (up to 140 mg/h) and then midazolam (up to 15 mg/h) was administered.

On day 3 of hospitalization, despite sedation and antiseizure medication (ASM), face and distal upper extremities myoclonic jerks continued to appear with similar intensity. Successive Neuron Specific Enolase (NSE) measurements at 48 hours (5.7 µg/L) and 72 hours (4.8 µg/L) were low (normal range: 0.0-16µg/L). Posterior EEGs did not show any epileptiform activity but due to the continued presence of myoclonic jerks levetiracetam was increased to 1.5 g every 12 hours and IV valproic acid 500 mg was added every 12 hours. Differential diagnoses of acute post-hypoxic status epilepticus, myxedema coma and hepatic encephalopathy were all excluded; thyrotropin (9.61 mUI/L, range 0.30-5) and ammonia levels (74 µmol/L, range <72) were slightly above the normal range and no other diagnostic criteria for the latter two entities were observed, nonetheless, the basal levothyroxine dose was increased from 75 µg/d to 100 µg/d, and lactulose and rifaximin were started.

On day 5 of hospitalization, the patient had normal exploration of negative 20 somatosensory evoked potentials (N20 SSEPs) and a brain magnetic resonance imaging (MRI) without any abnormalities. The same day midazolam was stopped with posterior increase in the frequency and intensity of myoclonic jerks, a new EEG did not show epileptiform activity, and midazolam and propofol were reintroduced.

On days 6 to 8 of hospitalization sedation with midazolam was stopped and propofol was gradually reduced while introducing clonazepam 1 mg every eight hours and perampanel 2 mg every day. Myoclonic jerks were still notable upon tactile and auditive stimulation, however a new EEG showed no EA. At this point the ASM regimen consisted of clonazepam 1 mg/8h, levetiracetam 1.5 g/12h, valproic acid 500 mg/12h and perampanel 2 mg/d.

On day 10, sedation with propofol was stopped and perampanel dose was increased to 6 mg/d. On the 11th day of the hospitalization the patient achieved a GCS of 6T (only normal flexion to pain, while intubated and on mechanical ventilation) for the first time since his ICU stay. A new brain MRI performed on the 15th day of hospitalization showed no acute lesions correlated to the patient’s clinical course. During the subsequent days the patient began to regain consciousness, achieving a GCS of 11T (obeying simple commands) on the 20th day of hospitalization, myoclonic jerks diminished but were still notable upon tactile and auditive stimulation and when the patient performed voluntary movements (Video 1). In this context we confirmed the diagnosis of LAS.

**Video 1:** Myoclonic jerks triggered by tactile and auditory stimulation Myoclonic jerks triggered by tactile and auditory stimulation in a Lance-Adams syndrome patient.

After regaining full consciousness, the patient became very cooperative and underwent vigorous physical and respiratory rehabilitation. The patient declined further aggressive intensive care treatments because of severe physical disabilities resulting from the prolonged illness and ICU stay. He was extubated after 28 days of admission and was posteriorly transferred to the Intermediate Care Unit. He was discharged to a rehabilitation center at day 50. Unfortunately, the patient was severely frail, myopathic and bedridden, unable to fully take care for himself, and continued to experience painful myoclonic jerks. Ten days after hospital discharge, the patient showed no more signs of improvement, and comfort sedation and analgesia were introduced as part of his palliative care. As a consequence of his progressive physical deterioration the patient died shortly after.

## Discussion

The physiopathology of LAS is poorly understood as the syndrome comprises diffuse damage to cortical and/or subcortical structures. It is hypothesized that myoclonic seizures arise from neurotransmitter abnormalities associated with gamma-aminobutyric acid (GABA) and serotonin neurotransmission [1,6,10-13]. Histopathological studies in LAS show that implicated structures of the brain include cerebellar vermian, paravermian structures, and diencephalus. However, differences in pathology could be due to the particular etiology of hypoxia or duration of the syndrome [1,14]. 

The most important clinical feature of LAS is the persistence of myoclonic jerks when consciousness returns [10], but deep sedation and even ASM may mask consciousness improvement and LAS may easily be confused with acute PHM [6,15]. The diagnosis of LAS includes the presence of postural and action myoclonic tremors, mainly in the face and upper arms, distal more than proximal. These myoclonic jerks are triggered by touch and sounds and disappear with relaxation and sleep. These may be exacerbated by the patient attempting to perform tasks, and their intensity is correlated with the required precision of said tasks [3,4,7,10]. Complementary tests may be necessary for the diagnosis of LAS or at least to discard acute PHM, since an incorrect diagnosis of acute PHM may lead to withdrawal of active medical treatment [4]. EEG is considered the most reliable tool for evaluating LAS and the most common finding is burst suppression [11]. Nonetheless, action myoclonus in LAS usually has no relationship to EEG and no EEG single pattern is specifically correlated with LAS [4,6,8,16]. According to Guo et al., one-third of patients with LAS showed EA, particularly within hours after cardiac arrest. Around 20% of patients have a normal EEG and 60% of patients show normal-sized giant SSEPs [9]. While different patterns may occur in time, the yield of standard intermittent EEG in cardiac arrest patients for outcome prognostication and identification of EA may not differ from the use of continuous EEG [17]. In summary, the absence of a pathological EEG does not rule out the possibility of LAS.

Specific brain damage biomarkers, such as NSE, and conventional imaging studies like computed tomography (CT) scans and magnetic resonance imaging (MRI), may not reveal any abnormalities in some cases [2,6,8]. For instance, a review of brain MRIs conducted on 12 LAS patients at an average of 2.5 years after the hypoxic event did not demonstrate any distinct abnormalities [9]. Complementary neuroimaging techniques, such as brain single-photon emission computed tomography (SPECT) or brain positron emission tomography (PET), have recently shown promising results in elucidating the anatomical and pathophysiological basis of LAS [10].

Our patient stopped receiving sustained sedation on the 10th day of hospitalization and fully regained consciousness until the 20th day, with varying levels of GCS in between, delaying the LAS diagnosis. The variable levels of sedation and ASM during the first 10 days of hospitalization may have masked an earlier return of consciousness in our patient. Initial differential diagnosis included acute PHM, hepatic encephalopathy, and myxedema coma; however, general blood and radiology studies did not support any of those diagnoses. Furthermore, despite the patient displaying indicators of an unfavorable neurological prognosis, notably including myoclonic jerks within the initial 24 hours post-cardiac arrest, our clinical decision involved the escalation of ASM pending the establishment of a conclusive diagnosis and posterior gradual de-escalation according to the clinical improvement of the patient as well as an absence of EA in the control EEGs.

Currently, the treatment approach for LAS is primarily based on proposed physiopathology, as there are no guidelines and no large controlled trials aimed at this purpose [1,6,18]. A study involving more than 100 patients with LAS revealed that clonazepam, valproate and piracetam were beneficial in approximately half of the cases [19]. Levetiracetam has demonstrated efficacy in improving myoclonus, while perampanel has been utilized in some case reports for difficult-to-treat LAS cases, without clear evidence [1,11,18,20].

In spite of the clinical findings in our patient, no complementary tests were definitive of irreversible neurologic damage and most failed to show abnormalities. MRI and CT scans did not show irregularities (we did not perform a PET scan). EEGs exhibited normal traces without EA. All these outcomes are consistent with the existing body of scientific literature [1,4,6,10,16]. In conjunction with ASM, physical therapy has shown the potential to slow the progression of LAS and reduce the risk of additional disabilities [6]. For refractory cases of LAS, deep brain stimulation surgery has been attempted with initially promising results; however, it remains an experimental treatment option [1].

Vital prognosis for patients with LAS is generally favorable and responds well to treatment with ASM [6], despite this, the condition significantly impairs the quality of life for affected individuals. In addition to action, myoclonus patients with LAS may experience epilepsy (30%), dysarthria (30%), and gait disturbance 70% [2]. A substantial proportion of patients are unable to independently carry out basic daily activities or return to work. Moreover, the presence of negative myoclonus or brief lapses in muscle tone predisposes these patients to frequent falls, often leading to their confinement to wheelchairs [2,4,6,7].

Our patient underwent a stepwise ASM, incorporating valproate, clonazepam, levetiracetam, and ultimately perampanel. Concurrently, intensive physical therapy was administered. However, despite achieving an accurate diagnosis and implementing an appropriate therapeutic and rehabilitation regimen, the patient remained profoundly debilitated and incapable of enduring the ramifications of an extended hospitalization and his incapacitating neurological condition. Discussions regarding the withdrawal of life-sustaining treatment were openly conducted both within the medical team and with the patient's family. Once the patient regained consciousness, he expressed his decision to decline further medical intervention unless there was a clear clinical improvement. Ultimately, the patient succumbed to his condition and passed away.

## Conclusions

LAS and acute PHM represent two distinct myoclonic syndromes, yet while LAS is characterized by the emergence of myoclonic jerks and cerebellar ataxia after the patient regains consciousness both syndromes may overlap at the beginning of the hypoxic insult. Distinguishing between the two conditions can be particularly challenging, especially in patients who are under sedation in the ICU, a problem that may lead to incorrect treatment or even withdrawing life-sustaining measures. The underlying pathophysiology of LAS is still inadequately understood and further research is needed to establish comprehensive guidelines and evidence-based approaches to effectively manage this syndrome. While the short-term prognosis of LAS is generally favorable, it is noteworthy that there is a paucity of available literature pertaining to the long-term prognosis or mortality. Nevertheless, it is well-established that LAS profoundly affects the quality of life for affected individuals, and a significant proportion of them endure severe disability.
